# Using whole-exome sequencing and protein interaction networks to prioritize candidate genes for germline cutaneous melanoma susceptibility

**DOI:** 10.1038/s41598-020-74293-5

**Published:** 2020-10-14

**Authors:** Sally Yepes, Margaret A. Tucker, Hela Koka, Yanzi Xiao, Kristine Jones, Aurelie Vogt, Laurie Burdette, Wen Luo, Bin Zhu, Amy Hutchinson, Meredith Yeager, Belynda Hicks, Neal D. Freedman, Stephen J. Chanock, Alisa M. Goldstein, Xiaohong R. Yang

**Affiliations:** 1 Division of Cancer Epidemiology and Genetics, National Cancer Institute, National Institutes of Health, Bethesda, MD 20892 USA; 2 Cancer Genomics Research Laboratory, Leidos Biomedical Research, Frederick National Laboratory for Cancer Research, Frederick, MD USA

**Keywords:** Cancer, Genetics, Systems biology

## Abstract

Although next-generation sequencing has demonstrated great potential for novel gene discovery, confirming disease-causing genes after initial discovery remains challenging. Here, we applied a network analysis approach to prioritize candidate genes identified from whole-exome sequencing analysis of 98 cutaneous melanoma patients from 27 families. Using a network propagation method, we ranked candidate genes by their similarity to known disease genes in protein–protein interaction networks and identified gene clusters with functional connectivity. Using this approach, we identified several new candidate susceptibility genes that warrant future investigations such as *NGLY1, IL1RN, FABP2, PRKDC*, and *PROSER2*. The propagated network analysis also allowed us to link families that did not have common underlying genes but that carried variants in genes that interact on protein–protein interaction networks. In conclusion, our study provided an analysis perspective for gene prioritization in the context of genetic heterogeneity across families and prioritized top potential candidate susceptibility genes in our dataset.

## Introduction

*CDKN2A* and *CDK4* are the two well-established high-risk genes for familial cutaneous malignant melanoma (CMM). In the last several years, *BAP1, POT1, ACD, TERF2IP*, and *TERT* were also identified as high-risk melanoma susceptibility genes^1^. Separately, intermediate-risk and low-risk genes have been identified, primarily from genome-wide association studies (GWAS). However, overall, mutations in known genes account for melanoma risk in less than 40% of melanoma-prone families, suggesting the existence of additional high-risk genes or perhaps a polygenic mechanism involving multiple genetic contributions^2^. Identifying additional high-risk melanoma susceptibility genes has been challenging because of the presence of extensive genetic heterogeneity, the rarity of recurrent mutations, and the complexity of the underlying genetic susceptibility^3^.

Although the application of Whole Exome Sequencing (WES) has been helpful in identifying potential disease-causing genes, confirming disease-causing variants after the initial discovery remains challenging. Even after all the filtering steps to remove common, low-impact, and non-cosegregating variants, there are usually multiple variants within a single family that are potentially related to the disease. In addition, variants in the same genes are rarely seen in multiple families (“private mutations”), requiring the development and adaptation of new analytical methods to address these issues. Here, we leverage a framework of the network analysis to identify candidate CMM genes that are connected at the functional level. Many genes exert their functions as components of protein complexes that represent molecular machineries, signaling pathways or cellular structures. Complicated molecular assemblies, however, do not necessarily fit the definition of conventional signaling pathways. Protein–protein interaction (PPI) networks, which represent the cellular network of all protein–protein interactions (interactome), may provide a powerful resource complementing genetic data to reveal complex interactions affected in disease states.

Gene prioritization methods leveraging interaction networks are based on the observation that genes related to similar diseases tend to lie close to one another in PPI networks^4^. Recently, a group of methods accounting for the global structure of networks have emerged to assess the proximity and connectivity between known disease genes (seeds) and candidate genes^5–7^. Central to network global methods is the common paradigm of network propagation, which is a powerful transformation method that can be applied to gene prioritization, gene function prediction, module discovery, disease characterization, and drug target prediction^8^. In recent years, computational approaches based on PPI networks have been successfully applied to interpret genomic data from WES and GWAS, and to identify molecular interactions affected by somatic mutations and germline variations in multiple diseases^9–14^.

**Figure 1:**
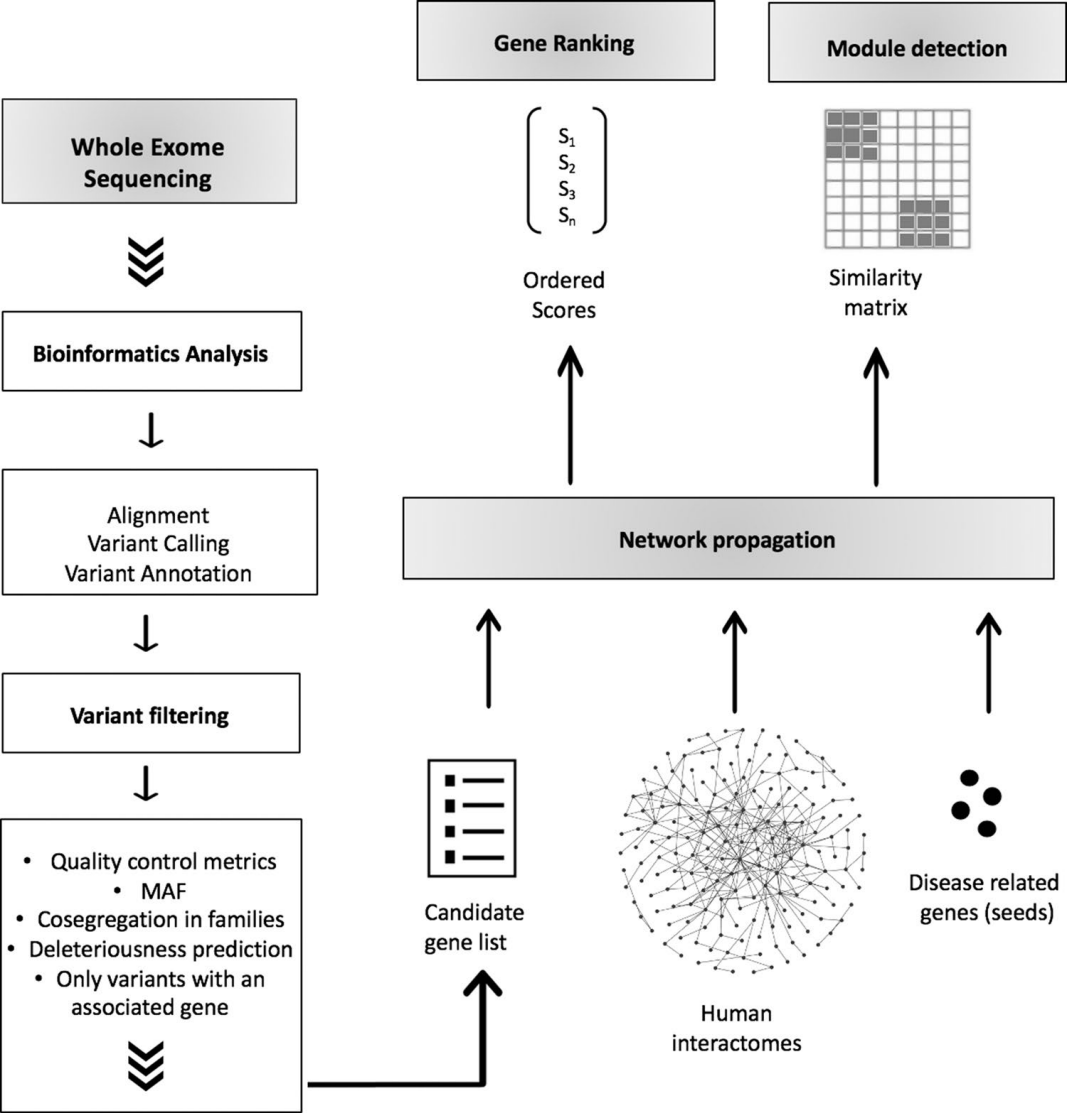
Methodological workflow. After Whole Exome Sequencing analysis of 27 CMM families, the resulting variants were filtered, and methods based on the network propagation principle were applied to prioritize candidate genes in the vicinity of genes previously related to CMM. Seed genes are known CMM genes (including susceptibility genes and somatic drivers) and candidate genes are those identified from the whole-exome sequencing analysis. The network propagation amplifies a biological signal based on the assumption that genes underlying similar phenotypes tend to interact with one another. Each gene was scored by its similarity to every other gene in protein interaction networks (interactomes). These scores or probabilities were then used to rank candidate genes and reveal gene clusters, respectively. Degree aware algorithm (DADA) was applied for gene ranking, and Hierarchical HotNet and GeneMANIA tools were used to identify modules. The variant filtering plan excluded variants based on population frequency in databases and internal controls, predicted pathogenicity, cosegregation in families, and quality control measures (see “Methods” section for filtering details).

In this study, we applied an analytic approach to integrate germline WES data with knowledge of the human PPI network architecture. Specifically, we applied network analyses based on the propagation principle, which incorporates previous knowledge of CMM susceptibility or driver genes (seed genes) to prioritize candidate genes identified from WES of CMM families (Fig. 1).

Using this approach, we nominated top candidate genes for further follow-up and identified gene networks that may relate to CMM susceptibility. Further, our study provides an analysis perspective on gene prioritization in the context of genetic heterogeneity across families.

## Methods

All family members who were willing to participate in the study provided written informed consent under a National Cancer Institute (NCI) Institutional Review Board (IRB) approved protocol (NCT00040352; 02-C-0211). All methods were performed in accordance with the relevant guidelines and regulations.

### Study population

All diagnoses of melanoma were confirmed by reviewing pathologic materials/reports, medical records, or death certificates. All study participants were of European ancestry from the United States. Originally the exome sequencing analysis included 144 melanoma cases from 76 families^2^. For this analysis, we selected 27 families (n = 98 CMM patients) with at least three sequenced CMM cases/obligate carriers within a family to enrich for genetic cases. The selected families in our analysis did not carry mutations in known high-risk CMM genes.

### Whole-exome sequencing and bioinformatics analysis

WES was performed at the Cancer Genomics Research Laboratory, National Cancer Institute (CGR, NCI). Details of the exome capture, WES, and bioinformatics pipeline used have been previously described^2,15,16^. Briefly, SeqCAP EZ Human Exome Library v3.0 (Roche NimbleGen, Madison, WI) was utilized for exome sequence capture. Exome sequencing was performed to a sufficient depth to achieve a minimum coverage of 15 reads in at least 80% of the coding sequence from the UCSC hg19 transcripts database. Variant discovery and genotype calling were performed globally using three variant callers (UnifiedGenotyper and HaplotypeCaller modules from GATK and FreeBayes [v9.9.2]). We included all target regions, as well as a 250-bp flanking region on each side. An Ensemble variant calling pipeline (v0.2.2) was then implemented to integrate the analysis results from the above mentioned three callers. Subsequently, the Ensemble variant calling pipeline that applies a Support Vector Machine (SVM) learning algorithm was used to identify an optimal decision boundary based on the variant calling results out of the multiple variant callers to produce a more balanced decision between false positives and true positives.

### Gene and variant filtering

Variants were included in the network analysis if they met the following criteria: (1) had a minor allele frequency (MAF) of < 0.001 in the 1000 Genomes Project, Exome Sequencing Project (ESP6500), and Exome Aggregation Consortium (ExAC); (2) were observed in ≤ 2 families from an in-house database (CGR, NCI) of ∼ 2000 exomes in ∼ 1000 cancer-prone families (excluding melanoma-prone or pancreatic cancer families); (3) were present in at least 3 sequenced CMM cases/obligate carriers within a family; (4) were classified as non-synonymous including frameshift, stop-gain, inframe deletion or insertion, missense, and splicing site variants; (5) were not located in highly variable genes; and (6) were likely to be deleterious for missense variants based on at least 2 of the 3 in silico predictions (Meta Likelihood ratio: D, METASVM: D, and CADD: ≥ 20). The first two algorithms are ensemble prediction scores that incorporate results from nine algorithms (SIFT, PolyPhen-2, GERP ++, Mutation Taster, Mutation Assessor, FATHMM, LRT, SiPhy, and PhyloP) and allele frequency^17^. Variants flagged with our pipeline quality control metric (CScorefilter), had a read depth < 10, ABHet < 0.2 or > 0.8, or were called by only one of the three callers used were excluded. Resulting variants were then aggregated into genes for the subsequent network analysis.

### Network-based candidate disease gene prioritization

We started with known genes with roles in CMM as the seeds and then applied the network propagation principle to prioritize/rank the group of candidate genes observed and selected from our exome analysis. Although the methods varied by how the propagation or diffusion was applied, the same propagation principle was common to all methods. The seed genes served as starting points for a random walk from node to node along the links of the network. At every step of the iterative algorithm, the current position moves to a randomly selected neighbor. After every move, the position is reset to a randomly chosen seed gene with a given probability (the restart value). After sufficient iterations, the frequency with which the nodes in the network are visited converges and is then used to rank the corresponding genes. Genes that are visited more often are considered to be closer to the seed genes and therefore are more relevant to the disease than those visited less often^18^.

We chose seed proteins that included known high-, intermediate- and low-risk genes for CMM, that had been identified primarily by family studies, linkage and GWAS^2^ (Table 1). Most of these loci involve genes and pathways that are known to play important roles in melanoma, such as telomere biology, cell cycle, pigmentation and nevi density. Our seed list also included genes considered to be somatic drivers for CMM, which were primarily compiled from The Cancer Genome Atlas (TCGA) analysis^19,20^ (see Table 1).

**Table 1:** Cutaneous malignant melanoma (CMM) related genes used as seeds by network propagation algorithms.

Genes	Risk^a^	Associated pathway/driver
ACD	High	Telomere
TPP1	High	Telomere
BAP1	High	Cell cycle
CDKN2A	High and low	Cell cycle
CDK4	High	Cell cycle
POT1	High	Telomere
TERF2IP	High	Telomere
IRF4	Intermediate	Pigmentation
MC1R	Intermediate	Pigmentation
MITF	Intermediate	Melanocyte differentiation
SLC45A2	Intermediate	Pigmentation
AGR3	Low	Unknown
ARNT	Low	Xenobiotic metabolism
ASIP	Low	Pigmentation
ATM	Low	DNA repair
CASP8	Low	Apoptosis
CCND1	Low	Cell cycle
CDKAL1	low	Unknown
CDKN2B	Low	Cell cycle
FTO	Low	DNA repair
HERC2	Low	Pigmentation
KITLG	Low	Pigmentation
MTAP	Low	9p21.3 locus
MX2	Low	Unknown
OBFC1	Low	Telomere
OCA2	Low	Pigmentation
PARP1	Low	DNA repair
PLA2G6	Low	Nevi
SLC24A5	Low	Pigmentation
TERT	Low & high	Telomere, Nevi
CLPTM1L	Low & high	Telomere, Nevi
RAD23B	Low	DNA repair
TMEM38B	Low	DNA repair
TYR	Low	Pigmentation
TYRP1	Low	Pigmentation
BRAF	Unknown	Driver
NRAS	Unknown	Driver
HRAS	Unknown	Driver
NF1	Unknown	Driver
RAC1	Unknown	Driver
MAP2K1	Unknown	Driver
TP53	Unknown	Driver
ARID2	Unknown	Driver
DDX3X	Unknown	Driver
PPP6C	Unknown	Driver
PTEN	Unknown	Driver
RB1	Unknown	Driver

^a^Disease-related genes included known high, intermediate, and low-risk genes for CMM identified by family studies, linkage, and GWAS^2^. Somatic drivers for CMM were also included^19,20^.

### Gene ranking

To rank genes, we applied the classic Random Walk with Restart (RWR) algorithm developed by Li and Patra^18^, and an improved version of it called DADA^21^. DADA, which fundamentally uses RWR, also provides statistical adjustment models to correct for ascertainment bias by accounting for the degree of connection among target genes since highly connected genes may be sensitive to the skewed distribution of PPI networks. To test our pipeline and strategy to identify relevant CMM genes, we included two families that carry variants in two well-known high-risk genes for CMM (POT1 and CDKN2A). We used the same filtering strategies and seed genes (with the exception of POT1 and CDKN2A) for the network analysis in these two positive-control families.

Permutation test was performed based on the outcome of the RWR algorithm applied to the interactomes using known CMM genes (Table 1) as seed nodes. According to genes yielded by the RWR algorithm, each node (gene) in the networks received a score representing its probability of being a potential risk gene. Genes with high probabilities are highly likely to be CMM risk genes. However, due to the topological structure of networks, some of the RWR resulting genes are not functionally related to CMM and are likely false-positive genes. Thus, to correct for the occurrence of false positive findings, a permutation test was performed to evaluate the probability of each candidate gene produced by RWR to be a significant CMM gene with several random gene sets used as seeds in comparison to actual ones. 1000 Ensembl ID sets with 47 genes (the number of total seeds originally applied) were randomly produced and each set was used as seed nodes. Then, each candidate gene received a probability value. After all 1000 sets were tested, each gene received one actual probability based on which a P-value was calculated as follows:

$${\text{P }} = \, \theta/{1}000;$$

where θ is the number of randomly produced sets in which the gene probability is larger than the probability computed by RWR using the original seed set from Table 1. We selected the value of 0.05 as the P-value threshold for controlling false-positive findings.

### Interactome sources

To evaluate the impact of different sources of interaction data, we used three interaction databases for the analysis of gene ranking and cluster detection algorithms: InWeb_IM network^22^, Reactome^23^, and HINT + HI^24,25^. The selected networks have differences in terms of protein interaction sources, validation methods and completeness, but all are considered high-quality interactomes and together represent a good representation of protein interaction in human cells

### Gene module detection

For module/subnetwork detection, we applied the Hierarchical HotNet^26^ and GeneMANIA algorithms^27,28^. Hierarchical HotNet identifies altered subnetworks or clusters containing genes that are both highly altered in a dataset and are topologically close on an interaction network. Hierarchical HotNet controls for ascertainment bias in the network by penalizing high degree nodes and also provides statistical significance testing. Hierarchical HotNet (i) combines network topology and vertex scores, (ii) defines a similarity matrix using a random walk-based approach, (iii) constructs a hierarchy of clusters consisting of strongly connected components, (iv) assesses the statistical significance of clusters in the hierarchy, (v) identifies altered clusters from statistically significant regions of the hierarchy and (vi) combines these clusters from multiple networks and sets of vertex scores. For the mutation score required by this method, each gene was assigned a score incorporating the percentage of patients who carry the mutation in our dataset. We also included the seed genes in our candidate list to increase the chances of finding interacting genes.

GeneMANIA was used as a plugin in Cytoscape^29^ with the candidate genes and seeds as input genes to map interactions and build a PPI network based on physical interactions. The GeneMANIA algorithm uses its own sources of interactions to places interacting genes into clusters and predicts new disease-related genes with their categorized functional association implied by multiple interaction datasets. The method consists of two parts: a linear regression-based algorithm that calculates a single composite functional association network based on multiple data sources (sources by default) and a label propagation algorithm that was used to predict gene function given the composite functional association network.

### Graphical layouts and analysis

Visualizations were performed with the layout algorithms in Gephi^30^ and Cytoscape software^31^.

### Enrichment analysis

For gene ontology enrichment, we used the functional enrichment component of GeneMANIA using the nodes that belong to the connected components, as these nodes may carry greater functional significance.

### Pedigree variant annotation, analysis, and search tool (pVAAST)

pVAAST was applied to obtain statistical evidence of disease-gene association. The software was used to perform gene/variant-based linkage analysis combined with functional prediction and rare variant case–control analysis in a family by family approach to evaluate the combined statistical evidence of disease-gene association^32^. We used WES data from 598 cancer-free controls from the Prostate, Lung, Colorectal, and Ovarian Cancer Screening Trial (PLCO) and Cancer Prevention Study (CPS) as reference/controls for the rare-variant association test. These controls, who were also of European ancestry, were sequenced and analyzed using the same sequencing platform and Ensemble variant calling pipeline as used for the familial CMM patients.

## Results

WES was conducted on 98 patients/obligate gene carriers in 27 CMM families without known mutations. After excluding variants based on quality of variant calls, population frequency, predicted pathogenicity, cosegregation in families (see “Methods” section for details of these filtering steps), a total of 364 variants in 360 genes (defined as candidate genes) were included in subsequent analyses. Only 10 of these genes had variants in two families, and none were observed in more than two families, highlighting the need for alternative approaches to prioritize candidate genes. Then, we applied the network propagation principle to rank candidate genes identified from our germline exome analysis using a group of genes previously associated with the disease designated as seeds genes (Table 1).

We used DADA to rank genes and three different interactome sources were used to evaluate the impact of different sources of network data on gene ranking. Table 2 shows genes consistently ranked high by DADA across the three networks with permutation P-values < 0.05 (probabilities from RWR are shown in S3 Table).

**Table 2:** Gene prioritization by the DADA algorithm.

Ranking	InWeb_IM^a^	P-value^d^	HIND + HI^b^	P-value^d^	Reactome^c^	P-value^d^
1	**ATM**	0.002	**ATM**	0.001	**TYR**	0.001
2	**CDKN2B**	0.003	**CDKN2B**	0.001	**ATM**	0.001
3	**TYR**	0.005	**CDKAL1**	0.001	**CDKN2B**	0.001
4	**CDKAL1**	0.001	**MAP2K2**	0.001	PRKCB	0.006
5	**PRKDC**	0.007	**PLCE1**	0.002	**MAP2K2**	0.005
6	**MLLT4**	0.037	**PRKDC**	0.008	**ATR**	0.034
7	**CD14**	0.034	**CD14**	0.041	ERBB2	0.024
8	**PLCE1**	0.011	**MLLT4**	0.03	FGFR3	0.043
9	EIF3A	0.017	**IL1RN**	0.008	IKZF3	0.005
10	**MAP2K2**	0.015	PKM	0.04	**ERCC3**	0.04
11	PHKB	0.006	**FABP2**	0.007	RNF4	0.006
12	**ATR**	0.024	CUL7	0.039	**MLLT4**	0.049
13	BIRC6	0.026	**ECI1**	0.024	FLT3	0.008
14	DAG1	0.027	**PROSER2**	0.02	TIMP1	0.009
15	**IL1RN**	0.007	**CYP4F11**	0.007	ARAP2	0.023
16	**ERCC3**	0.022	**CUL9**	0.012	MYT1	0.009
17	ARHGAP8	0.039	**NGLY1**	0.015	**NGLY1**	0.02
18	**FABP2**	0.019	CASZ1	0.029	TNFRSF10D	0.033
19	DCAF11	0.05	NR4A2	0.031	DDI2	0.009
20	CDC42BPG	0.015	VPS13D	0.043	MAP3K6	0.031
21	**PROSER2**	0.013	SALL4	0.038	PRAM1	0.016
22	ANKS1A	0.047	GOLGA6B	0.037	PTPRO	0.001
23	**ECI1**	0.011	IL22	0.037	PTPN5	0.039
24	**NGLY1**	0.023	PRR5	0.027	CYP3A7	0.042
25	**CYP4F11**	0.008	PRDM9	0.002	BTN2A1	0.046
26	CD93	0.037	CYP7A1	0.004	OMA1	0.021
27	**SPTLC2**	0.035	KCNU1	0.002	KLK12	0.045
28	UTP20	0.039	GRM8	0.002	**SPTLC2**	0.038
29	WDR5B	0.035	CALCA	0.009	ESYT1	0.019
30	**CUL9**	0.03	ACOT4	0.003	DUOX2	0.013

Genes ranked by two or more interactomes are highlighted in bold.

^a^InWeb_IM network consists of high-quality and scored protein interactions aggregated from 8 source databases^22^.

^b^HINT + HI corresponds to binary and co-complex interactions in HINT^24^ with high-throughput derived interactions from the HI network^25^.

^c^Reactome integrates several large-scale experimental data sets to build and train a machine-learning system that identifies potential functional interactions among pairs of human proteins^23^.

^d^Permutation P-values after applying RWR.

Rankings of candidate genes were consistent across the three networks. Top genes identified included both known CMM genes (*ATM*, *CDKN2B, TYR,* and *CDKAL1*) and genes that were previously unknown in CMM susceptibility, such as *PRKDC, MLLT4, PLCE1, MAP2K2, IL1RN,* and *ATR.* As a proof of principle analysis for the utility of DADA in gene prioritization, we applied the same ranking strategy in two additional CMM families with mutations in known CMM genes (*POT1* and *CDKN2A*). Using the same filtering and seed-gene selection scheme (excluding *POT1* and *CDKN2A* as seeds) as for the main analysis, we identified *POT1* and *CDKN2A* as the top ranked gene in each of these two families, respectively (S1 Fig).

**Figure 2:**
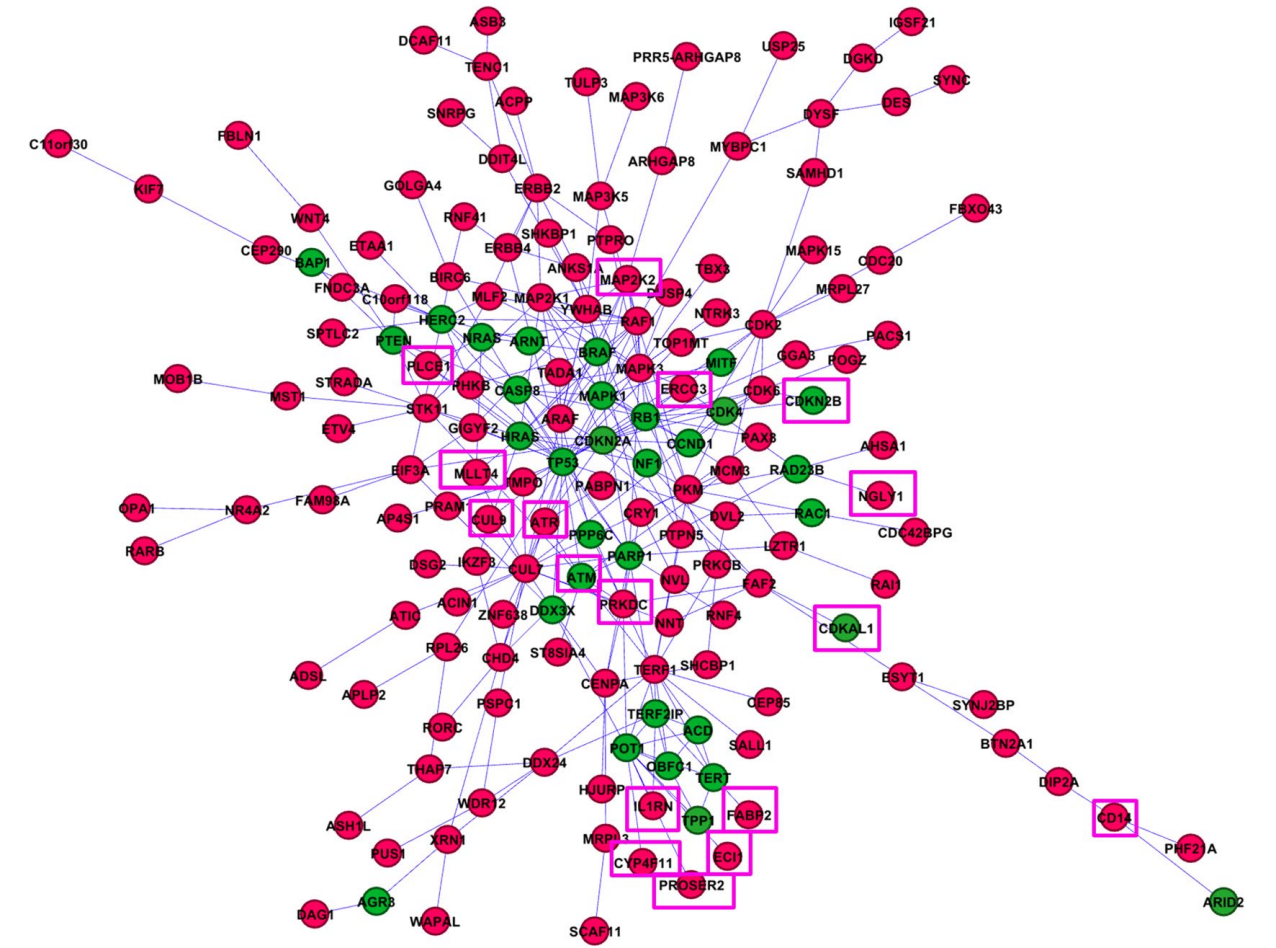
Interconnected genes module. Figure shows the cluster of interacting proteins identified by GeneMANIA. Genes/proteins are prioritized based on their interactions with proteins previously associated with the disease. Seeds: green; Candidate genes from exome sequencing analysis of familial melanoma data: red. The top genes prioritized by DADA are shown in squares.

We then used the GeneMANIA tool to identify modules of interconnected proteins that have direct protein–protein interactions with seeds. We found 315 protein–protein interactions between 34 seeds (driver and susceptibility genes) and the 360 candidates from our WES analysis. 72% of all seed genes from Table 1 were mapped to an interconnected cluster demonstrating that CMM driver/susceptibility genes are highly interconnected. About half of our candidate genes were also mapped to this highly interconnected cluster (Fig. 2). We focused on genes in the interconnected module in further prioritization steps since these genes may have stronger functional relevance compared to genes not found in network clusters.

Almost all top genes ranked by DADA were also mapped to the interconnected cluster (squared in Fig. 2), demonstrating a high consistency of results across different network approaches. In addition to ranking, the interconnected module also allows a visualization of the complex relationships between seed and candidate genes at the functional level. Importantly, some candidate genes in the module showed direct interactions with the most relevant seeds (known high-risk CMM genes), such as *ATR* and *EIF3A* (with *CDKN2A*), and *PRKDC*, *PROSER2*, and *IL1RN* (with *POT1*). We identified 13 direct interactions between candidate genes and high-risk CMM susceptibility genes (Table 3). In addition, we also identified some direct interactions between candidate genes and CMM driver genes, such as *PLCE1,* and *MLLT4* (with *NRAS/HRAS*), *MAP2K2* (with *BRAF*) (S1 Table).

**Table 3:** Known high-risk cutaneous malignant melanoma (CMM) genes and their first interacting neighbors identified in the interconnected gene cluster by GeneMANIA.

Melanoma high risk gene	Interacting partner
ACD	TERT
	OBFC1
	POT1
	TERF1
	TERF2IP
TPP1	POT1
	TERT
BAP1	PTEN
CDKN2A	**GGA3**
	**ATR**
	CCND1
	CDK4
	CDK6
	**EIF3A**
	HRAS
	MAP2K1
	TP53
CDK4	CDKN2A
	CCND1
	**CDKN2B**
	**PKM**
	RB1
	CDK6
POT1	ACD
	DDX3X
	**PROSER2**
	**IL1RN**
	**PRKDC**
	TERF2IP
	**ECI1**
	**CYP4F11**
	OBFC1
	TPP1
	TERT
	TERF1
TERF2IP	**DDX24**
	**FABP2**
	**IL1RN**
	ACD
	OBFC1
	PARP1
	TERF1

Genes in bold are found in our candidate gene list from WES analysis.

S2 Figure depicts the Degree Score, reflected by the number of interactions between one gene and other genes in the network. *TP53* and *CUL7* showed the highest centrality scores indicating the importance of these genes for the network structure from the topological analysis. We also performed a Gene Ontology (GO) enrichment analysis including all genes in the network identified by GeneMANIA and found significant enrichment for categories that are related to protein serine/threonine kinase activity, telomere complex, cell aging, and cell cycle (S2 Table).

**Figure 3:**
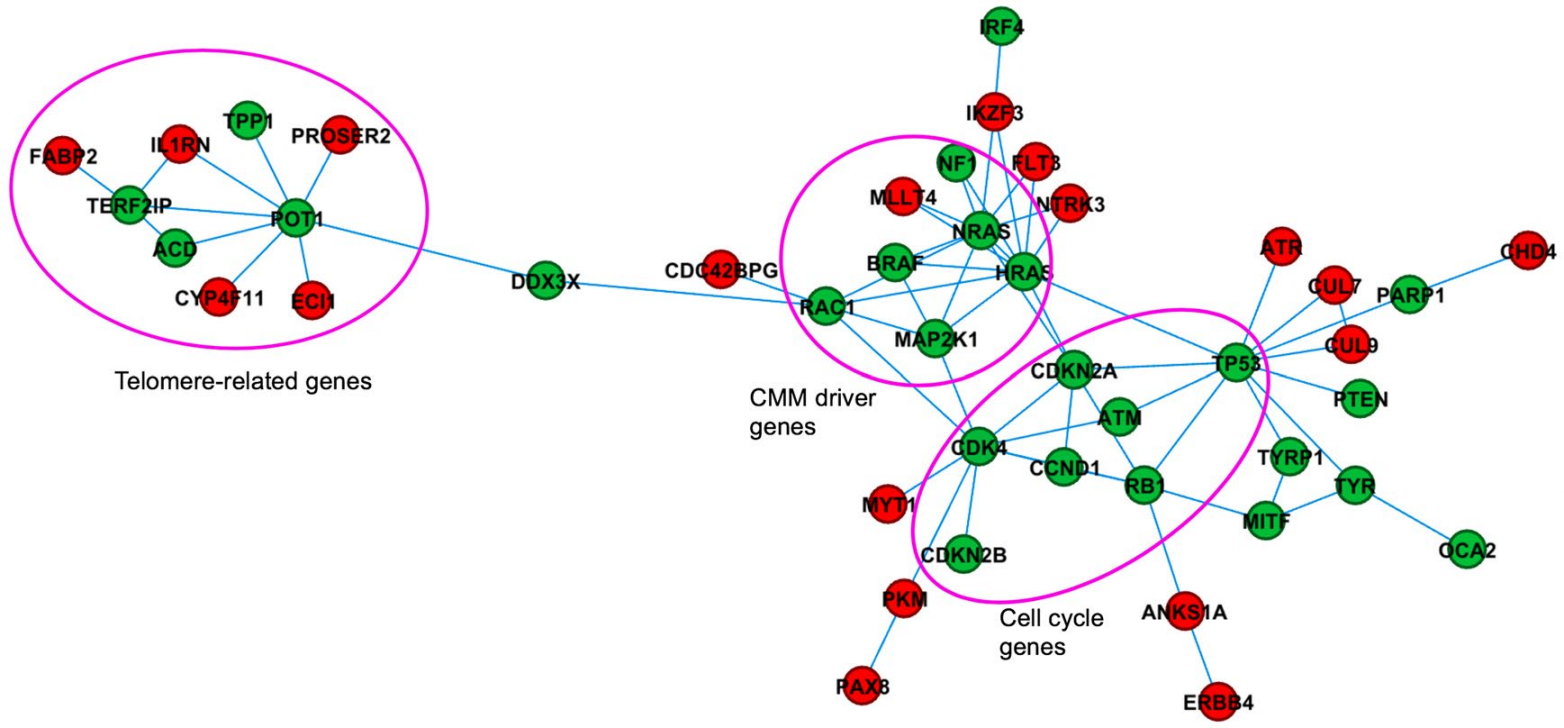
Hierarchical HotNet consensus subnetwork. Green circles indicate known melanoma genes and red circles indicate candidate genes from our candidate genes list, many of these genes were also ranked high by DADA and found by GeneMANIA. Genes that are part of biological processes such as telomere biology, cell cycle, or mutated in tumors are circled.

We also used a different module analysis approach, the hierarchical HotNet consensus algorithm, to identify significantly altered subnetworks/modules containing genes that are both altered and topologically close on interaction networks. In total, hierarchical HotNet analysis recovered 25 known CMM genes (Fig. 3, green circles) and 20 interacting partners as novel potential CMM genes (red circles) across three integrated interactomes, forming three groups of conglomerates corresponding to functions associated with telomere biology, cell cycle, and somatic drivers. Telomere genes showed a clear separation from the other groups, consisting of four known CMM genes (*POT1, ACD, TPP1, TERF2IP*) and several potential candidate genes such as *PROSER2, ECI1, IL1RN, CYP4F11* and *FABP2,* which were also ranked high by DADA. Overall, hierarchical HotNet analysis detected a smaller number of interacting genes compared to GeneMANIA. Several genes/interactions were identified by both analyses, including the telomere related genes. Most of these new candidate genes were also ranked high by DADA, suggesting a high confidence in functional connectivity across these genes.

We applied a Pedigree Variant Annotation, Analysis, and Search Tool (pVAAST), a unified test of linkage, functional prediction, and rare-variant association, to further prioritize genes with statistical evidence. Table 4 summarizes variants in genes ranked high by DADA and/or genes that showed high interactions with seed genes by GeneMANIA or HotNet. In particular, several of these genes also showed strong evidence for disease association using pVAAST (*IL1RN, NGLY1, FABP2, PROSER2, *and* CDC42BPG). *These genes are therefore considered to be some of the most plausible CMM candidate genes in our dataset.

**Table 4:** Gene variants identified by network analysis.

Gene	Chr	Location	SNP ID	REF	VAR	Variant type	Freq in family^a^	Family ID	Outgroup count^b^	MAF in control dataset			Variant impact	Pathogenecity prediction^c^			pVAAST	pVAAST	Prioritization method^f^	P-value^g^
										EXAC	ESP	1 KG		METASVM	METALR	CADD	P-value^d^	Rank^e^		
IL1RN	2	113890242	rs757992723	A	C	Missense	3/5	FF2	0	4.50E−05			Moderate	T	T	24.6	3.03E−04	2	DADA/GM^S^/HH^S^	0.007
ERCC3	2	128046437	rs200443230	T	A	Missense	3/5	A2	0	l.50E−05			Moderate	T	T	21	ns		DADA/GM^D^	0.022
ATR	3	142232392	rs200070057	T	C	Missense	3/5	A2	0	2.55E−04	3.49E−04		Moderate	T	T	25.5	ns		DADA/GM^SD^/HH^D^	0.024
NGLY1	3	25775422	rs201337954	T	A	Stop_gained	3/4	T	0	2.70E−04	2.33E−04		High				8.00E−06	1	DADA/GM^S^	0023
FABP2	4	120241839	Rs367603528	C	T	Missense	3/3	A6	0	l.51E−05			Moderate	T	T	30	2.74E−04	1	DADA/GM^S^/HH^S^	0.019
CD14	5	140012379	rs151227107	G	C	Missense	3/5	B22	1	3.78E−04	9.30E−04		Moderate	T	D	12.74	ns		DADA/GM^D^	0.034
MLLT4	6	168312131	rs769690450	G	A	Missense	3/3	A6	0	4.50E−05			Moderate	T	T	21.9	6.03E−03	98	DADA/GM^D^/HH^D^	0.037
MLLT4	6	168348545	rs773338292	G	C	Missense	3/5	A2	0	1.50E−05			Moderate	T	T	25.2	ns		DADA/GM^D^/HH^D^	0.037
ANKS1A	6	34985418	rs748921780	C	T	Missense	4/5	A2	0				Moderate	T	T	24.4	3.03E−04	13	GM^D^/HH^D^	0.047
PRKDC	8	48840360	rs35938758	C	T	Missense	3/5	FF2	0	2.48E−04	1.19E−04		Moderate	T	T	25.8	ns		DADA/GM^SD^	0.007
EIF3A	10	120796765	rs367880512	G	A	Missense	4/4	F10	0	4.50E−05	1.16E−04		Moderate	T	T	21.6	4.50E-04	13	GM^SD^	0.017
PLCE1	10	95931182		T	G	Missense	3/3	X	0				Moderate	T	T	26.3	ns		DADA/GM^D^	0.011
PROSER2	10	11908784	rs779142603	T	C	Splicing	3/3	D2	0	3.17E−05			High			12.08	4.11E−04	7	DADA/GM^S^/HH^S^	0.013
CDC42BPG	11	64597710	rs150779995	A	G	Missense	3/3	D2	0				Moderate	T	T	28.4	3.52E−04	2	GM^D^/HH^D^	0.015
ECI1	16	2294529	rs375300423	C	T	Missense	4/5	FF2	1	2.53E−04	1.16E−04		Moderate	D	D	17.19	5.09E−04	14	DADA/GM^S^/HH^S^	0.011
CYP4F11	19	16025439	rs200031770	G	C	Stop_gained	3/4	F10	0	8.99E−05	1.16E−04		High			13.01	ns		DADA/GM^S^/HH^S^	0.008
MAP2K2	19	4101254		G	A	Stop_gained	3/5	FF2	0				High			17.68	ns		DADA/GM^D^	0.015

*Chr* chromosome, *REF* reference allele, *VAR* variant allele, *Freq* frequency, *CMM* cutaneous malignant melanoma, *MAF* minor allele frequency, *T* tolerant, *D* deleterious, *RWR* random walk with restart.

^a^Number of cases with the variant/number of cases sequenced in this family.

^b^Internal family controls: ~ 2000 exomes from ~ 1000 cancer families (excluding melanoma or pancreatic cancer families).

^c^Pathogenicity prediction for missense variants based on in silico algorithms, METALR and METASVM, which are ensemble prediction scores that incorporate results from nine algorithms and allele frequency.

^d^*pVAAST* Pedigree Variant Annotation, Analysis, and Search Tool. Gene/variant-based linkage analysis combined with functional prediction and rare variant case–control analysis to evaluate the combined statistical evidence of disease-gene association in each family; *ns* non-statistically significant.

^e^pVAAST rank: Candidate genes were ranked based on P-values from the combined pVAAST test.

^f^Prioritization method: *GM* GeneMANIA, *HH* Hierarchical HotNet; *DADA* Degree-Aware Disease Gene Prioritization Algorithm; ^S^protein interacting with susceptibility seed; ^D^protein interacting with driver seed.

^g^Permutation P-values after RWR algorithm and InWeb_IM network.

**Figure 4:**
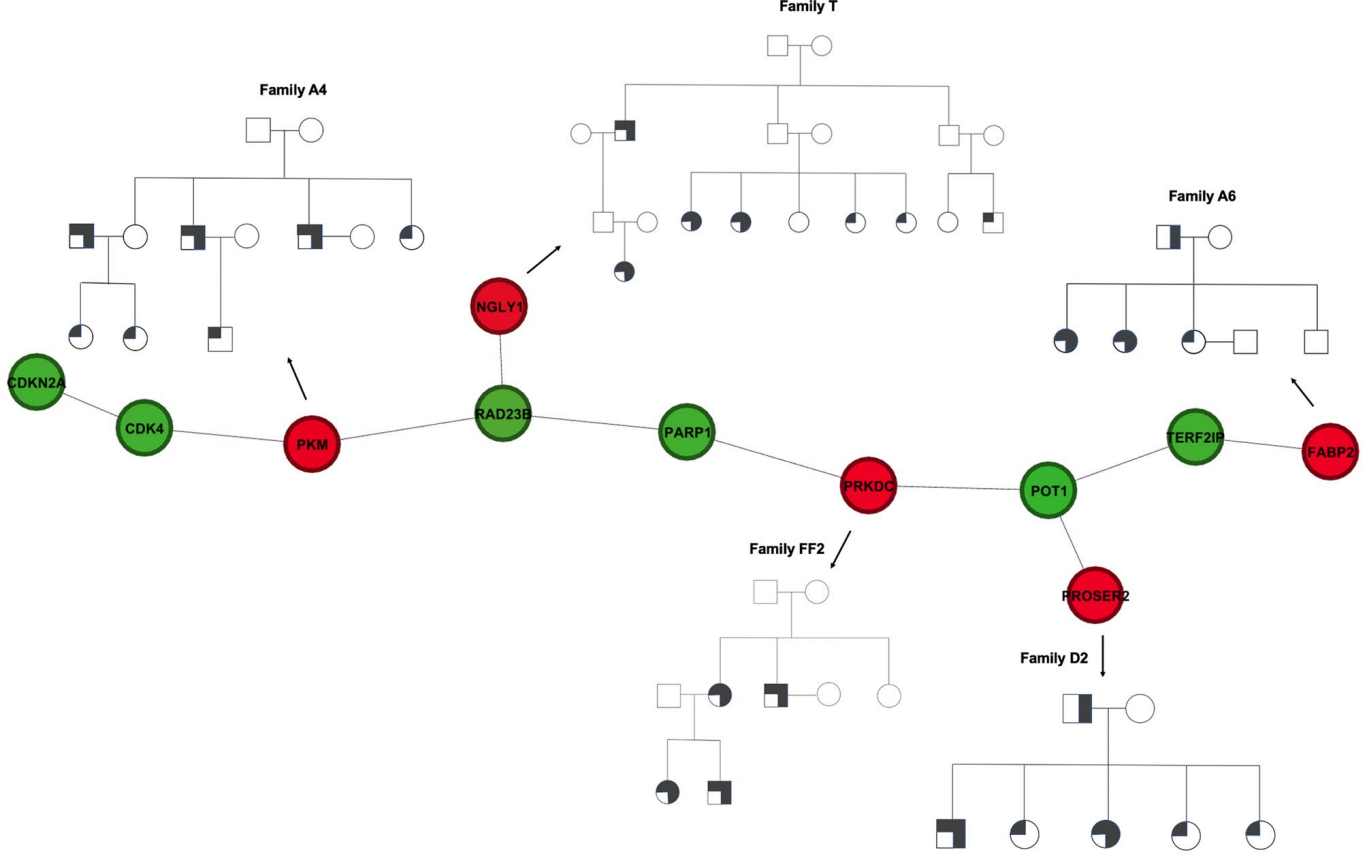
Dissection of protein–protein interactions. Families are connected by genes that show interactions on PPI networks. Only prioritized genes and their direct connections with seeds are shown, recovered through a manual examination of the GeneMANIA module. Green circles indicate known melanoma genes and red circles indicate candidate genes. Solid squares and circles: CMM cases; Circles: females; Squares: males.

We present an example to illustrate how network analyses could be helpful in gene identification when each studied family has a distinct set of top candidate genes. The analysis was performed with reconstructed interactions by GeneMANIA. In a pedigree (Family T) with four sequenced CMM patients, we identified a stop-gain variant in *NGLY1* that was carried by all four cases as well as an obligate gene carrier (subject 1008, Fig. 4). In contrast, only one unaffected family member carried the variant. The stop-gain variant (c.1201A > T; p.R401X) was determined as the top variant in this family by pVAAST (p = 8.00E−06) (Table 4). While this gene would be considered a strong candidate, no variants in *NGLY1* were seen in any other families examined. Through network propagation, we found that *NGLY1* interacts directly with *RAD23B* (seed), a low-risk CMM gene. Following the flow of interactions, *RAD23B* is directly connected to *PKM* and indirectly connected to *PRKDC* (through *PARP1*) and *PROSER2* (through *POT1*). Missense variants in *PKM* and *PRKDC* were carried by all sequenced cases in Family A4 and three cases in FF2, respectively, while a variant in the splicing region of *PROSER2* was seen in all three sequenced cases in family D2 (Fig. 4). These results suggest that network propagation may link families that do not share variants in the same affected gene but involve genes that interact with each other in a PPI network. In summary, our results allowed for gene prioritization from an extensive list of gene candidates, detection of novel genes associated with modules with functional relevance, and clustering of families carrying affected genes in close proximity.

## Discussion

It remains challenging to identify novel disease genes with WES because of the large number of candidate mutations, the small number of available patients/families for variant intersection, and the complexity of susceptibility itself. Our study, applying a network analysis approach in combination with a family-based statistical test for linkage/association, may provide a methodological framework to investigate genetic heterogeneity and propose a focused candidate gene list for functional characterization. Using this approach, we identified *NGLY1, IL1RN, FABP2, PRKDC*, and *PROSER2* as the strongest candidate genes in our familial CMM dataset. Specifically, we were able to link families with “private” gene mutations based on interconnectivity of these variants in PPI networks.

Network propagation from seed genes allowed us to prioritize potential new susceptibility genes previously obscured within the large list of variants/genes identified from WES evaluation. In our WES analysis, 360 genes remained on the candidate gene list after all filtering steps, which would pose a serious challenge to subsequent functional evaluation work. Using DADA, we ranked genes based on their interactions with known melanoma genes and identified several top genes that were previously unknown in CMM susceptibility. The top ranked genes were confirmed to interact in gene modules with important seeds. Most of these top candidate genes have important biological functions that are relevant in cancer development or related processes. For example, *PRKDC* encodes a serine/threonine DNA-PKc that is a molecular sensor of DNA damage and is involved in the ligation step of the non-homologous end joining (NHEJ) pathway of DNA double strand break (DSB) repair^33^. *PRKDC* also interacts with telomeres influencing chromosome end integrity, dynamics^34^ and the risk of CMM in melanoma‐prone families^35^. The observation that CMM susceptibility genes encode highly interconnected modules may at least partially explain the observed genetic heterogeneity of CMM, i.e., variants in any member of the molecular module may lead to similar functional alterations that subsequently contribute to risk.

The module detection analysis strategy resulted in inclusion of genes, for which no direct evidence of involvement was previously available, that have close interactions with known CMM genes in the same clusters (e.g.; *PROSER2, IL1RN,* and *FABP2* in the telomere biology cluster). *PROSER2* is also known as *C10orf47*, for which the function is largely unknown. *FABP2* encodes a fatty acid-binding protein that regulates liposynthesis and global metabolism^36^. In addition, proteins in the *FABP* family are thought to play a role in gene regulation, cell signaling, cell growth and differentiation^37^, and alterations in this gene have been reported in different types of cancers. For example, *FABP7* expression was found to be associated with tumor progression in melanoma^38^. *IL1RN*, which was also found interacting with telomere proteins in our network analysis, encodes a member of the interleukin 1 cytokine family and modulates a variety of immune and inflammatory responses. *IL1RN* polymorphisms have been associated with cancer susceptibility^39^ and clinical prognosis in melanoma patients with aggressive disease^40^. The role of these genes in telomere biology has not been previously reported. Here, we found immediate interactions between these genes and known high-risk CMM genes (*POT1* and *TERF2IP*) involved in telomere maintenance, using the information from a high-resolution map of the telomere interactome in living human cells, a method that is capable of detecting even low- affinity or transient interactions^41^. Most of the interactions represented by the interactomes come from large-scale screening studies that offer a reliable source of information, including tandem affinity purification and yeast two-hybrid experiments. Notably, these genes also showed strong statistical evidence for association/linkage and were ranked high by pVAAST, further highlighting the need for investigating their functions in relation to CMM susceptibility.

The underlying principle of propagation addressed the evident genetic heterogeneity by detecting genes that are not necessarily present in multiple families but co-occur in close proximity to the propagated network. Using this principle, we identified families that did not share variants/genes in common but were connected through a similar molecular landscape. For example, *NGLY1* is the strongest candidate gene in one of our most informative families. The stop-gain variant (c.1201A > T; p.R401X) is a reported pathogenic variant (ClinVar accession: VCV000050962); compound heterozygous or homozygous genotype of this allele caused *NGLY1* deficiency autosomal recessive disorder of the Endoplasmic Reticulum-Associated Degradation (ERAD) pathway (PMID: 24651605). In family T, this mutant allele, which was present in the heterozygous state, showed complete co-segregation with disease with suggestive high penetrance (only one examined unaffected family member harbored the allele) and was ranked as the top gene by pVAAST. However, rare non-synonymous variants in *NGLY1* were not observed in any other families sequenced. Through network analysis, however, we were able to connect the *NGLY1* family with several other families that carried variants in genes interacting with each other in PPI networks. Among them, *PROSER2* and *FABP2* are connected through telomere genes as previously mentioned. *PRKDC* was ranked as top genes by DADA and showed direct interactions with both known CMM susceptibility genes and melanoma driver genes. Given the important functions of these genes and the interconnectivity among them, these genes should be considered potential candidates and followed up in further genetic and functional evaluations.

The analytical approach used in this study may also help examine the relationships between germline and somatic variants since we included germline susceptibility and somatic driver genes as seeds. For example, it is worth exploring the role of *DDX3X* (driver gene), which appeared connecting a group of drivers with a cluster of high-risk genes (Fig. 3). We also evaluated somatic nonsynonymous mutations in genes prioritized by our network analyses in melanoma samples included in The Cancer Genomic Atlas (TCGA). Somatic mutations in genes prioritized in our study were common in TCGA, which were seen in ~ 43% of all tumors (S3 Fig), suggesting potential biological relevance. These mutations did not vary significantly across different genomic subtypes^20^.

Despite the increasing and successful applications of interaction networks in scientific discoveries, some limitations need to be considered. First, incompleteness (false negatives) and noise (false positives), which are the two inherent problems of the available network sources, may affect the gene prioritization work. To address this issue, we used three different interactomes and focused on genes that were ranked high in multiple analyses. Second, the current network algorithms do not provide formal statistical testing to evaluate the significance of a given propagation score. Further, rankings should be used for gene prioritization rather than for determining causality. To address this limitation, we also used a family-based association/linkage analysis approach, pVAAST and a permutation test after RWR to provide statistical evidence for candidate genes identified by the network algorithms. Third, current interaction networks are static in that they were not created across multiple time points or under a particular cellular context. Furthermore, the network approach may not be applicable in diseases for which the causal or susceptibility genes do not interact with previously known proteins or when there is little information on known disease genes as seeds. Another limitation not restricted to network strategies is that candidate variants may be regulatory or structural and would not be identified by WES analysis and therefore would not be found using interaction network approaches.

In summary, we applied a network analysis perspective to prioritize candidate genes by integrating variant analysis with the protein–protein interaction network architecture. Using this approach, we identified plausible genes that may be associated with CMM susceptibility in our high-risk CMM-prone families. The results demonstrate the value of a network propagation principle through seed proteins in gene prioritization. Further evaluation of the top identified candidate genes is needed to determine their importance in melanoma susceptibility.

## Supplementary information

Supplementary Information.

## Data Availability

The dataset generated during and/or analyzed during the current study are available from the corresponding author on reasonable request.
