# Supplementary material for: Using whole-exome sequencing and protein interaction networks to prioritize candidate genes for germline cutaneous melanoma susceptibility
Source: Sci Rep. 2020 Oct 14;10:17198. doi: 10.1038/s41598-020-74293-5 (PMC7560829; doi:10.1038/s41598-020-74293-5)
Supplement: Supplementary file 1 — Supplementary Information. [file 41598_2020_74293_MOESM1_ESM.docx]

**Using whole-exome sequencing and protein interaction networks to prioritize candidate genes for germline cutaneous melanoma susceptibility**

Sally Yepes^1^*, Margaret A. Tucker^1^, Hela Koka^1^, Yanzi Xiao^1^, Kristine Jones^1,2^, Aurelie Vogt^1,2^, Laurie Burdette^1,2^, Wen Luo^1,2^, Bin Zhu^1,2^, Amy Hutchinson^1,2^, Meredith Yeager^1,^ ^2^, Belynda Hicks^1,2^, Neal D. Freedman^1^, Stephen J. Chanock^1^, Alisa M. Goldstein^1,3^, Xiaohong R. Yang^1,3^ *

^1^Division of Cancer Epidemiology and Genetics, National Cancer Institute, National Institutes of Health, Bethesda, MD, 20892, USA

^2^ Cancer Genomics Research Laboratory, Leidos Biomedical Research, Frederick National Laboratory for Cancer Research, Frederick, MD, USA

^3^Co-senior authors

*Corresponding authors

[royang@mail.nih.gov](mailto:royang@mail.nih.gov), sally.yepestorres@nih.gov

**
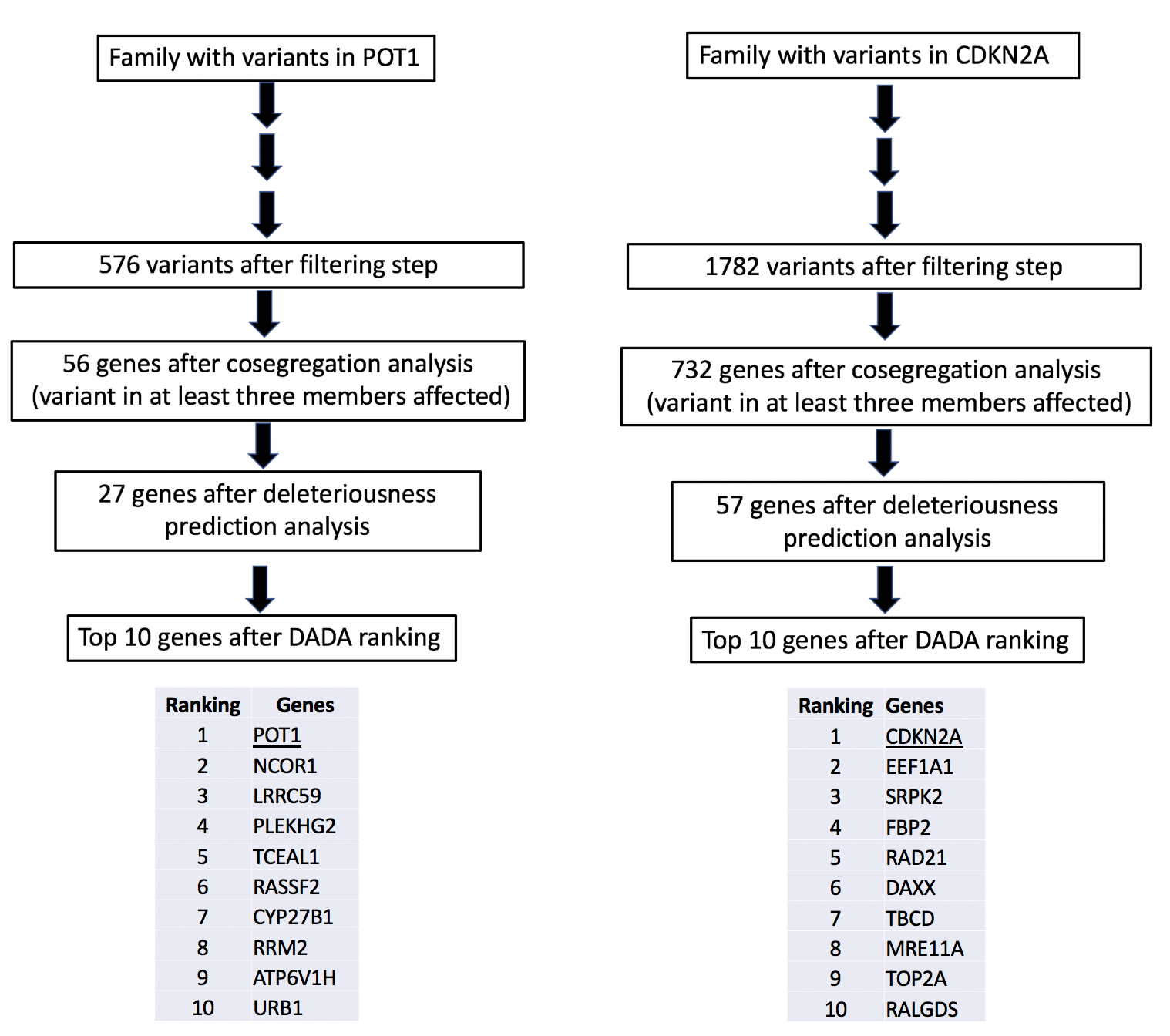
**

**S1 Fig. Variant prioritization pipeline in families carrying well-known susceptibility genes.**

**
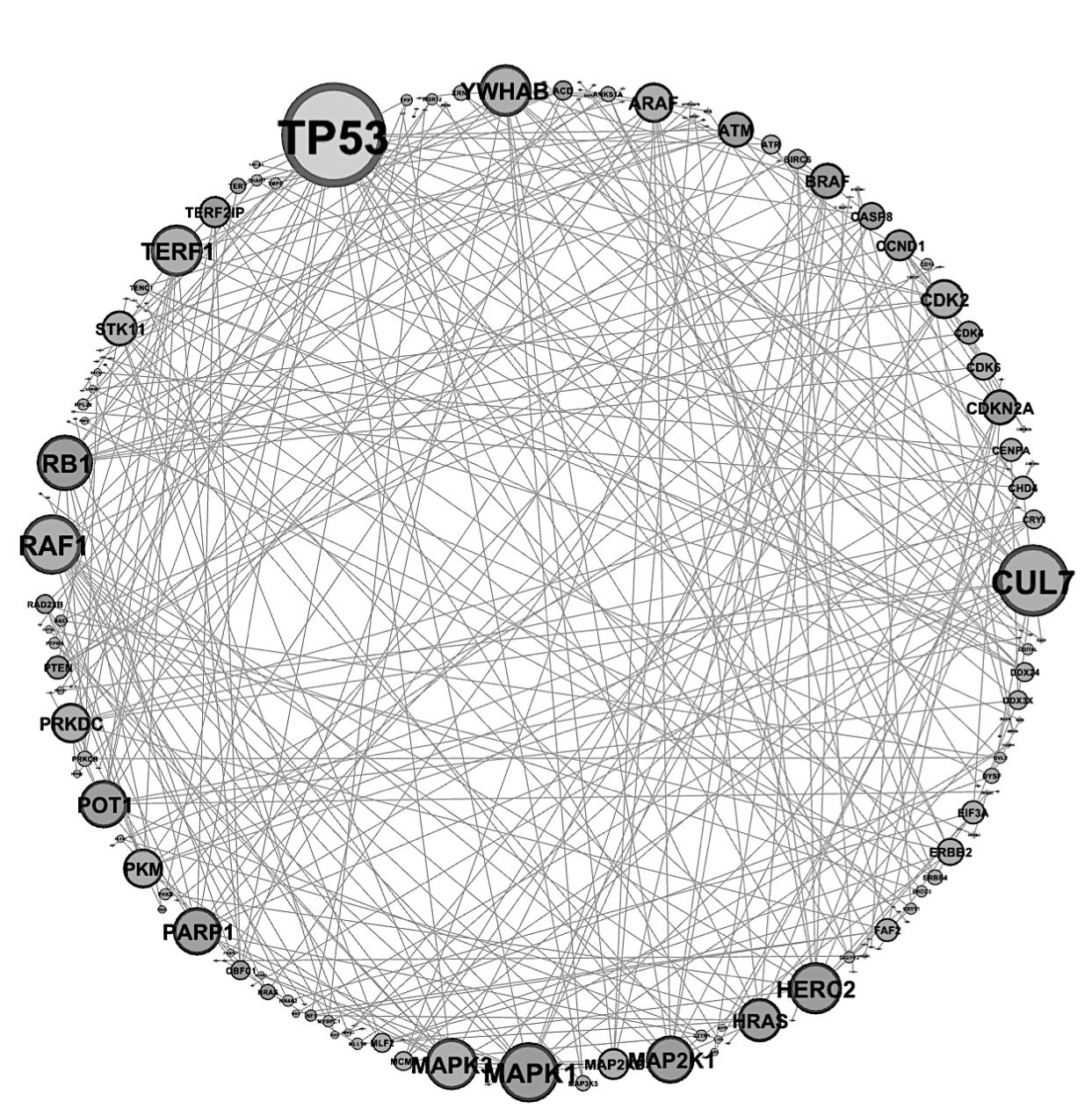
**

**S2 Fig. Degree centrality.** Figure shows the genes with the highest centrality measure by node degree (the number of other nodes that are directly connected to a particular node), where the greater the node degree value the larger the size of the circle.

**
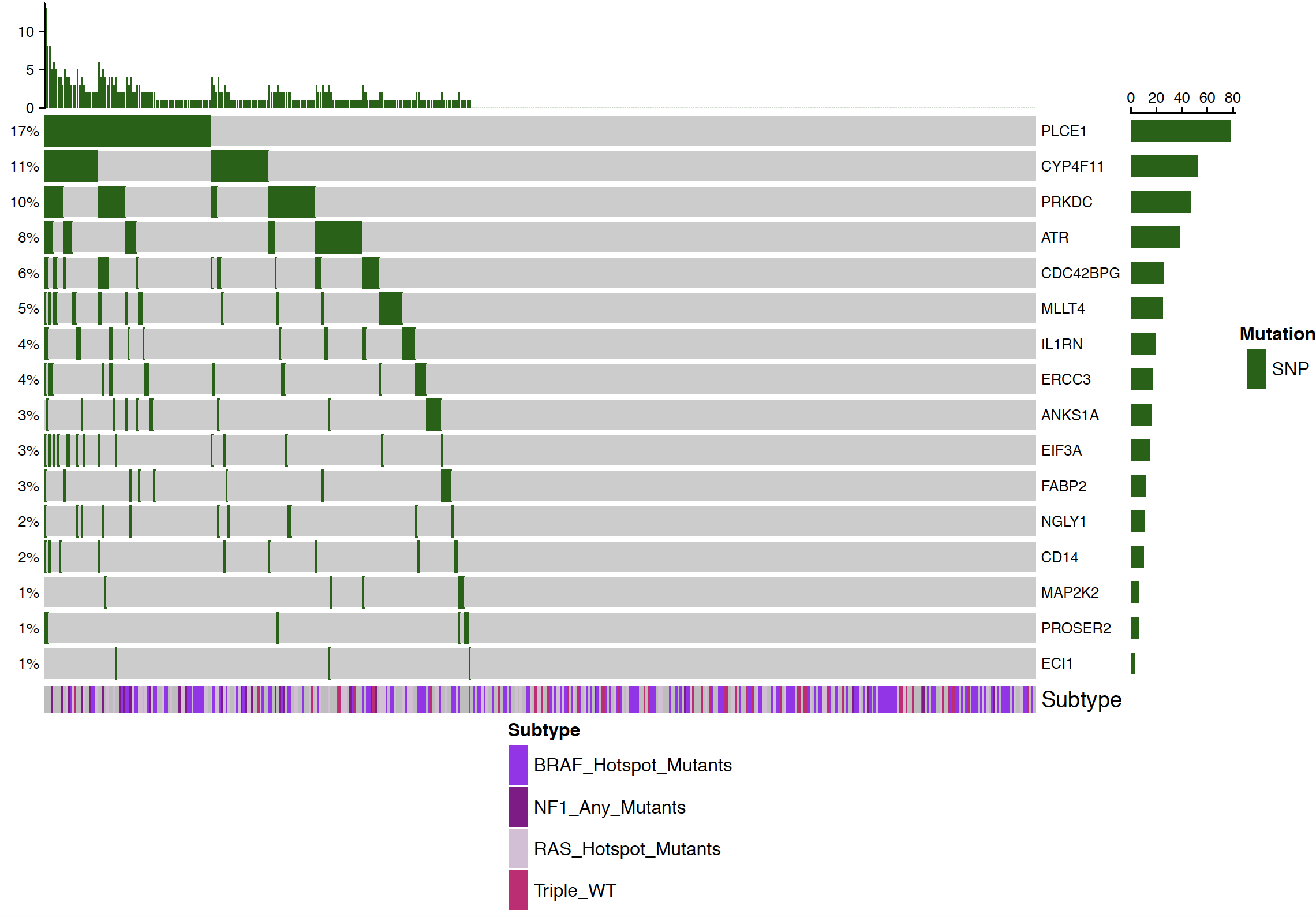
**

**S3 Fig. Oncoprint of somatic alterations in genes prioritized by network analyses**.

**S1 Table. CMM driver genes and their first interacting neighbors identified in the interconnected gene cluster.**

| **Driver gene** | **Interacting partner** |
| --- | --- |
| BRAF | ARAF |
|  | HRAS |
|  | MAP2K1 |
|  | **MAP2K2** |
|  | MAPK1 |
|  | NRAS |
|  | **PHKB** |
|  | RAF1 |
| NRAS | BRAF |
|  | **MLLT4** |
|  | **PLCE1** |
|  | RAF1 |
| HRAS | ARAF |
|  | BRAF |
|  | CDKN2A |
|  | MAP2K1 |
|  | **MLLT4** |
|  | NF1 |
|  | **PLCE1** |
|  | RAF1 |
|  | TP53 |
| NF1 | HRAS |
| RAC1 | **CDC42BPG** |
| MAP2K1 | ARAF |
|  | BRAF |
|  | HRAS |
|  | MAPK1 |
|  | RAF1 |
| TP53 | **ATM** |
|  | **ATR** |
|  | CASP8 |
|  | CDKN2A |
|  | **CUL9** |
|  | DDX3X |
|  | **ERCC3** |
|  | HERC2 |
|  | HRAS |
|  | MAPK1 |
|  | PARP1 |
|  | **PHKB** |
|  | **PRKDC** |
|  | PTEN |
|  | RB1 |
|  | **TOP1MT** |
| ARID2 | **CD14** |
| DDX3X | EIF3A |
|  | POT1 |
|  | TP53 |
| PPP6C | ARAF |
|  | **PRKDC** |
| PTEN | BAP1 |
|  | CASP8 |
|  | TP53 |
| RB1 | **ANKS1A** |
|  | ARNT |
|  | CASP8 |
|  | CCND1 |
|  | CDK4 |
|  | CDK6 |
|  | MAPK1 |
|  | PABPN1 |
|  | RAF1 |
|  | TP53 |

**S2 Table.** Gene Ontology (GO) enrichment.

**S3 Table. Permutation P-values and probabilities of association based on RWR method**

| **Ranking** | **INWEB** | **Probability** | **P** | **Ranking** | **HIND+HI** | **Probability** | **P** | **Ranking** | **REACTOME** | **Probability** | **P** |
| --- | --- | --- | --- | --- | --- | --- | --- | --- | --- | --- | --- |
| 1 | **ATM** | 1.63E-02 | 0.002 | 1 | **ATM** | 1.68E-02 | 0.001 | 1 | **TYR** | 2.38E-02 | 0.001 |
| 2 | **CDKN2B** | 1.64E-02 | 0.003 | 2 | **CDKN2B** | 1.69E-02 | 0.001 | 2 | **ATM** | 1.97E-02 | 0.001 |
| 3 | **TYR** | 1.64E-02 | 0.005 | 3 | **CDKAL1** | 1.66E-02 | 0.001 | 3 | **CDKN2B** | 1.96E-02 | 0.001 |
| 4 | **CDKAL1** | 1.63E-02 | 0.001 | 4 | **MAP2K2** | 5.52E-04 | 0.001 | 4 | PRKCB | 3.79E-04 | 0.006 |
| 5 | **PRKDC** | 1.26E-04 | 0.007 | 5 | **PLCE1** | 1.27E-03 | 0.002 | 5 | **MAP2K2** | 2.84E-04 | 0.005 |
| 6 | **MLLT4** | 1.46E-04 | 0.037 | 6 | **PRKDC** | 1.94E-04 | 0.008 | 6 | **ATR** | 1.46E-04 | 0.034 |
| 7 | **CD14** | 1.61E-04 | 0.034 | 7 | **CD14** | 1.98E-04 | 0.041 | 7 | ERBB2 | 1.69E-04 | 0.024 |
| 8 | **PLCE1** | 2.43E-04 | 0.011 | 8 | **MLLT4** | 9.73E-05 | 0.03 | 8 | FGFR3 | 1.16E-04 | 0.043 |
| 9 | EIF3A | 1.31E-04 | 0.017 | 9 | **IL1RN** | 3.02E-04 | 0.008 | 9 | IKZF3 | 2.08E-04 | 0.005 |
| 10 | **MAP2K2** | 1.13E-04 | 0.015 | 10 | PKM | 9.65E-05 | 0.04 | 10 | **ERCC3** | 1.06E-04 | 0.04 |
| 11 | PHKB | 2.31E-04 | 0.006 | 11 | **FABP2** | 1.83E-04 | 0.007 | 11 | RNF4 | 2.50E-04 | 0.006 |
| 12 | **ATR** | 1.34E-04 | 0.024 | 12 | CUL7 | 1.19E-04 | 0.039 | 12 | FLT3 | 1.33E-04 | 0.008 |
| 13 | BIRC6 | 1.73E-04 | 0.026 | 13 | **ECI1** | 1.14E-04 | 0.024 | 13 | TIMP1 | 1.21E-04 | 0.009 |
| 14 | DAG1 | 1.81E-04 | 0.027 | 14 | **PROSER2** | 1.27E-04 | 0.02 | 14 | ARAP2 | 9.54E-05 | 0.023 |
| 15 | **IL1RN** | 2.54E-04 | 0.007 | 15 | **CYP4F11** | 3.03E-04 | 0.007 | 15 | MYT1 | 1.94E-04 | 0.009 |
| 16 | **ERCC3** | 1.49E-04 | 0.022 | 16 | **CUL9** | 2.14E-04 | 0.012 | 16 | **NGLY1** | 2.00E-04 | 0.02 |
| 17 | ARHGAP8 | 9.14E-05 | 0.039 | 17 | **NGLY1** | 2.90E-04 | 0.015 | 17 | TNFRSF10D | 1.03E-04 | 0.033 |
| 18 | **FABP2** | 1.91E-04 | 0.019 | 18 | CASZ1 | 1.44E-05 | 0.029 | 18 | DDI2 | 4.19E-04 | 0.009 |
| 19 | DCAF11 | 5.80E-05 | 0.05 | 19 | NR4A2 | 1.13E-05 | 0.031 | 19 | MAP3K6 | 9.28E-05 | 0.031 |
| 20 | CDC42BPG | 1.27E-04 | 0.015 | 20 | VPS13D | 6.42E-06 | 0.043 | 20 | PRAM1 | 3.16E-05 | 0.016 |
| 21 | **PROSER2** | 9.77E-05 | 0.013 | 21 | SALL4 | 7.00E-06 | 0.038 | 21 | PTPRO | 3.82E-04 | 0.001 |
| 22 | ANKS1A | 5.96E-05 | 0.047 | 22 | GOLGA6B | 5.49E-06 | 0.037 | 22 | PTPN5 | 1.20E-05 | 0.039 |
| 23 | **ECI1** | 1.09E-04 | 0.011 | 23 | IL22 | 7.49E-06 | 0.037 | 23 | CYP3A7 | 7.58E-06 | 0.042 |
| 24 | **NGLY1** | 1.31E-04 | 0.023 | 24 | PRR5 | 8.33E-06 | 0.027 | 24 | BTN2A1 | 9.67E-06 | 0.046 |
| 25 | **CYP4F11** | 1.66E-04 | 0.008 | 25 | PRDM9 | 7.95E-06 | 0.002 | 25 | OMA1 | 7.46E-06 | 0.021 |
| 26 | CD93 | 1.31E-05 | 0.037 | 26 | CYP7A1 | 7.19E-06 | 0.004 | 26 | KLK12 | 6.42E-06 | 0.045 |
| 27 | **SPTLC2** | 7.32E-05 | 0.035 | 27 | KCNU1 | 9.49E-06 | 0.002 | 27 | **SPTLC2** | 8.93E-06 | 0.038 |
| 28 | UTP20 | 7.95E-06 | 0.039 | 28 | GRM8 | 7.72E-06 | 0.002 | 28 | ESYT1 | 7.91E-06 | 0.019 |
| 29 | WDR5B | 7.46E-06 | 0.035 | 29 | CALCA | 7.69E-06 | 0.009 | 29 | DUOX2 | 7.00E-06 | 0.013 |
| 30 | **CUL9** | 3.90E-05 | 0.03 | 30 | ACOT4 | 7.99E-06 | 0.003 | 30 | ASIC4 | 7.55E-06 | 0.027 |
